# Creating an African HIV Clinical Research and Prevention Trials Network: HIV Prevalence, Incidence and Transmission

**DOI:** 10.1371/journal.pone.0116100

**Published:** 2015-01-20

**Authors:** Anatoli Kamali, Matt A. Price, Shabir Lakhi, Etienne Karita, Mubiana Inambao, Eduard J. Sanders, Omu Anzala, Mary H. Latka, Linda-Gail Bekker, Pontiano Kaleebu, Gershim Asiki, Ali Ssetaala, Eugene Ruzagira, Susan Allen, Paul Farmer, Eric Hunter, Gaudensia Mutua, Heeran Makkan, Amanda Tichacek, Ilene K. Brill, Pat Fast, Gwynn Stevens, Paramesh Chetty, Pauli N. Amornkul, Jill Gilmour, The IAVI Africa HIV Prevention Partnership

**Affiliations:** 1 Medical Research Council/Uganda Virus Research Institute, Uganda Research Unit on AIDS, Entebbe, Uganda; 2 International AIDS Vaccine Initiative (IAVI), New York, New York, United States of America; 3 Department of Epidemiology and Biostatistics, University of California San Francisco, San Francisco, California, United States of America; 4 Zambia-Emory HIV Research Project, Lusaka and Copperbelt, Zambia; 5 Project San Francisco, Kigali, Rwanda; 6 Centre for Geographic Medicine-Coast/KEMRI, Kilifi, Kenya; 7 University of Oxford, Oxford, United Kingdom; 8 Kenya AIDS Vaccine Institute—Institute of Clinical Research, Nairobi, Kenya; 9 Aurum Institute, Rustenburg and Johannesburg, South Africa; 10 Desmond Tutu HIV Centre, University of Cape Town, Cape Town, South Africa; 11 Emory University, Atlanta, Georgia, United States of America; 12 IAVI, Johannesburg, South Africa; 13 IAVI Human Immunology Laboratory, Imperial College, London, United Kingdom; 14 University of Alabama at Birmingham, Birmingham, Alabama, United States of America; 15 IAVI-UVRI HIV Vaccine Program, Entebbe and Masaka, Uganda; University of Washington, UNITED STATES

## Abstract

HIV epidemiology informs prevention trial design and program planning. Nine clinical research centers (CRC) in sub-Saharan Africa conducted HIV observational epidemiology studies in populations at risk for HIV infection as part of an HIV prevention and vaccine trial network. Annual HIV incidence ranged from below 2% to above 10% and varied by CRC and risk group, with rates above 5% observed in Zambian men in an HIV-discordant relationship, Ugandan men from Lake Victoria fishing communities, men who have sex with men, and several cohorts of women. HIV incidence tended to fall after the first three months in the study and over calendar time. Among suspected transmission pairs, 28% of HIV infections were not from the reported partner. Volunteers with high incidence were successfully identified and enrolled into large scale cohort studies. Over a quarter of new cases in couples acquired infection from persons other than the suspected transmitting partner.

## Introduction

The African continent is disproportionately affected by the HIV pandemic. Despite representing only 12% of the world’s population, sub-Saharan Africa bears nearly 70% of the world’s cases of HIV [1] and the epidemic there is complex [2]. The greatest diversity of HIV subtypes is found across Africa, primarily subtypes A, C, D and their recombinants, and this varies by region [3]. Modes of transmission and affected populations are equally diverse, and can change over time. While the epidemic is primarily heterosexual [4], other key populations including men who have sex with men [5] may also contribute to HIV transmission regionally. Policy makers and clinical trialists depend on epidemiology data to guide effective prevention planning, service delivery [6, 7] and design and conduct of prevention trials [2, 8, 9].

HIV surveillance systems have often relied on cross sectional surveys of HIV infection [10]. While prevalence data may provide some indication of risk groups and can provide guidance for service delivery, they fail to identify recently infected persons. Though there is some progress being made on assays to estimate incidence from prevalent samples [11], these assays have yet to be proven valid, particularly in the African context [12, 13]. Furthermore, the increasing availability of antiretroviral therapy throughout Africa is allowing persons with HIV to live longer, making prevalence data even less relevant [14]. Research towards understanding and preventing HIV transmission should ideally focus on incident cases of HIV. Large-scale HIV incidence cohorts remain the gold standard for collecting these data.

Beginning in 1999 the International AIDS Vaccine Initiative (IAVI) undertook the funding and development of in-country laboratory and clinical capacity at nine research centers in Kenya, Uganda, Rwanda, Zambia and South Africa to support HIV vaccine trials. This work included observational epidemiology studies designed to recruit key populations suitable for trials, provide critical experience to the research teams, and inform the design of future HIV prevention trials. In 2004 this network began multiple, large-scale studies of HIV prevalence, incidence and early infection. Here we present the findings of these observational epidemiology studies.

## Methods

### Clinical Research Network

**Figure 1:**
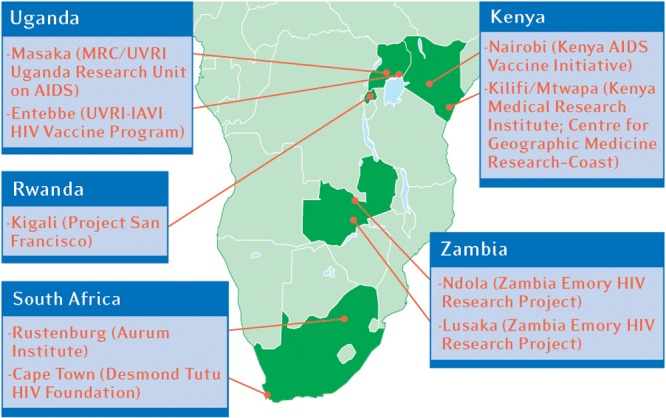
Map of the collaborating research centers of the IAVI Africa HIV Prevention Partnership. MRC/UVRI: Medical Research Council/Uganda Virus Research Institute, IAVI: International AIDS Vaccine Initiative

This work was conducted at nine sites in South Africa, Zambia, Kenya, Uganda and Rwanda (Fig. 1). All clinical research centers (CRC) are affiliated with existing institutions, locally staffed and most are led by local clinician-scientists who are recognized experts on HIV. To ensure that studies are conducted according to the International Conference on Harmonization Good Clinical Practices (ICH-GCP), GCP training is provided. HIV testing and counseling training and support are provided to counseling staff through a continuous improvement process. Specialized training addresses the needs of key populations such as men who have sex with men [15] or married/cohabiting couples [16, 17]. Study clinics are equipped to provide some care to volunteers (e.g., diagnosis and treatment of sexually transmitted diseases) and local health care resources available for referral have been assessed and strengthened where possible [18].

### Laboratory facilities

All laboratories are equipped to perform tests for HIV antibody (rapid test for HIV, HIV ELISA, and p24 ELISA), syphilis and pregnancy. To assure assay comparability across CRC, quality assurance and control (QA/QC) is performed by Clinical Laboratory Services in Johannesburg, South Africa. Laboratory personnel are trained in GCLP, and 8 of the nine research centers in the network have now received GCLP accreditation through Qualogy (www.qualogy.co.uk). The Rustenburg CRC did not adopt PBMC processing facilities, instead sending sample to Johannesburg for processing, and therefore never qualified for GCLP accreditation. Three labs were accredited in 2007 (Nairobi, Kilifi, Entebbe), one in 2008 (Kigali), one in 2009 (Lusaka), one in 2010 (Ndola) and one in 2011 (Masaka).

### HIV prevalence studies

Three CRCs (Nairobi, Masaka and Kilifi) enrolled 1,000–2,000 study volunteers, 18 years or older, for a single visit to characterize local HIV prevalence and risk factors. HIV VCT employed two rapid tests (typically Determine and Unigold; with tiebreakers Murex in Masaka, Vironostika in Nairobi, and Instascreen in Kilifi). A brief, site-specific, demographic and risk assessment questionnaire was administered. In Nairobi, volunteers included sex workers and their clients. In Masaka, the goal was to obtain a population-based estimate of prevalence and therefore recruitment included all residents of three rural communities, recruited and provided with HIV counseling and results at their homes. Home based counseling included pre-test counseling and sample draw in the home, with delivery of results within 1–4 weeks. Subsequent testing and counseling (in the incidence studies, below) was done in a local research clinic. In Kilifi, study volunteers were recruited from three populations: 1) randomly selected residents of the Kilifi Demographic Surveillance System (DSS), 2) walk-in VCT clients at the study site, and 3) the referred sexual partners of DSS and walk-in volunteers.

### HIV incidence studies

Methods for each cohort have been published separately (Table 1). Briefly, each CRC performed community mapping, community-sensitization exercises, and/or prevalence studies, and based on the results and their own area(s) of expertise, each team selected risk screening criteria to enroll key populations. Teams reviewed enrollment criteria and HIV incidence in real time to confirm enrollment of true key populations. The cohorts included: 1) HIV negative partners of co-habiting, sexually active couples with an HIV infected, antiretroviral therapy (ART)-naïve partner (Zambia; Rwanda; Entebbe, Uganda) or irrespective of ART status (Masaka, Uganda); 2) sexually active sex workers and their clients (Kenya); 3) members of fishing communities aged 13–49 years and reporting at least one of the following in the past 3 months: more than one partner, a new partner, a sexually transmitted infection (STI), absence from home for more than 2 nights, regular sex partner who is HIV infected (Uganda; in January 2012, criteria were modified to ages 18–49 years, ART-naïve, report of alcohol/recreational drug use, report of unprotected sex; more than one partner was dropped); 4) Men who have sex with men (MSM; Kenya and Rustenburg); 4) younger participants recruited in Cape Town (≥16 years) and in the first fishing community study (Uganda, ≥13 years); and 5) geography as a screening criterion, as in the recruitment of adult members of several study villages (Masaka) and women living in peri-urban, economically disadvantaged regions with high HIV prevalence (Rustenburg).

**Table 1:** HIV prevalence and multivariate predictors of prevalent HIV infection in Masaka, Kilifi and Nairobi.

	**Masaka**							**Kilifi**							**Nairobi Total**						
	**Total**		**HIV**					**Total**		**HIV**					**Total**		**HIV**				
**Characteristic**	**N**	**%**	**N**	**%**	**PR**	**p value**	**95% CI**	**N**	**%**	**N**	**%**	**PR**	**p value**	**95% CI**	**N**	**%**	**N**	**%**	**PR**	**p value**	**95% CI**
Total	1663	100	187	11.2	--			1769	100	146	8.3	--			996	100	163	16.4	--		
Volunteer sex																					
Male	650	39.1	56	8.6	ref.			726	41.0	39	5.4	ref.			296	29.7	20	6.8	ref.		
Female	1013	60.9	131	12.9	1.80	0.001	(1.28, 2.53)	1043	59.0	107	10.3	1.65	0.007	(1.15. 2.38)	700	70.3	143	20.4	3.05	<0.001	(1.95, 4.78)
Volunteer age (years)																					
18–24	425	25.6	30	7.1	ref.			596	33.7	29	4.9	ref.			287	28.8	14	4.9	ref.		
25–29	311	18.7	29	9.3	1.34	0.230	(0.83, 2.18)	356	20.1	38	10.7	1.75	0.016	(1.11, 2.76)	221	22.2	37	16.7	3.38	<0.001	(1.89, 6.06)
30–34	261	15.7	43	16.5	2.39	<0.001	(1.55, 3.69)	326	18.4	32	9.8	1.58	0.065	(0.97, 2.56)	150	15.1	36	24.0	4.83	<0.001	(2.71, 8.62)
35+	666	40.0	85	12.8	1.99	0.001	(1.33, 2.97)	491	27.8	47	9.6	1.37	0.183	(0.86, 2.19	338	33.9	76	22.5	4.44	<0.001	(2.58, 7.65)
Marital status																					
Single, never married	274	16.5	30	10.9		NS		461	26.1	18	3.9	ref.			521	52.3	68	13.1		NS	
Divorced or widowed	239	14.4	53	22.2				167	9.4	43	25.7	3.56	<0.001	(2.02, 6.29)	385	38.7	84	21.8			
Married	1150	69.2	104	9.0				1141	64.5	85	7.4	NA	--	--	90	9.0	11	12.2			
Married monogamous	NA	--	--	--				985	55.7	64	6.5	1.13	0.646	(0.66, 1.94)	NA	--	--	--			
Married polygamous	NA	--	--	--				156	8.8	21	13.5	2.27	0.011	(1.21, 4.28)	NA	--	--	--			
Location of home village																					
Distal from paved road	1286	77.3	128	10.0	ref.			NA	--	--	--				NA	--	--	--			
Adjacent to paved road	377	22.7	59	15.6	1.54	0.004	(1.15, 2.06)	NA	--	--	--				NA	--	--	--			
Location of current residence																					
Rural	NA	--	--	--				171	9.7	4	2.3	ref.			NA	--	--	--			
Urban	NA	--	--	--				1598	90.3	142	8.9	4.06	0.005	(1.52, 10.85)	NA	--	--	--			
Source of study volunteers																					
Randomly selected from community	NA	--	--	--				821	46.4	60	7.3		NS		NA	--	--	--			
Walk-in VCT clients	NA	--	--	--				315	17.8	35	11.1				NA	--	--	--			
Partners of above volunteers	NA	--	--	--				633	35.8	51	8.1				NA	--	--	--			
Reported condom use^*^																					
None of the time	1127	67.8	114	10.1	ref.			NA	--	--	--				NA	--	--	--			
Half or more of the time	167	10.0	17	10.2	1.06	0.809	(0.66, 1.71)	NA	--	--	--				NA	--	--	--			
Less than half of the time	369	22.2	56	15.2	1.54	0.006	(1.13, 2.08)	NA	--	--	--				NA	--	--	--			
Never	NA	--	--	--				1095	61.9	96	8.8		NS		NA	--	--	--			
Sometimes	NA	--	--	--				342	19.3	21	6.1				NA	--	--	--			
Always	NA	--	--	--				108	6.1	6	5.6				NA	--	--	--			
Not asked	NA	--	--	--				224	12.7	23	10.3				NA	--	--	--			
No	NA	--	--	--				NA	--	--	--				163	16.4	17	10.4	ref.		
Yes	NA	--	--	--				NA	--	--	--				833	83.6	146	17.5	1.64	0.036	(1.03, 2.59)
Sexually transmitted diseases reported in past 12 months (Masaka) or ever (Kilifi, Nairobi)																					
None	1000	60.1	85	8.5	ref.			1100	62.2	56	5.1	ref.			NA	--	--	--			
Urethral discharge	180	10.8	16	8.9	0.91	0.725	(0.55, 1.51)	556	31.4	73	13.1	1.24	0.202	(0.89, 1.72)	NA	--	--	--			
Genital ulcers	264	15.9	40	15.2	1.57	0.010	(1.11, 2.22)	280	15.8	55	19.6	2.48	<0.001	(1.79, 3.43)	NA	--	--	--			
Both ulcers and discharge	219	13.2	46	21.0	2.16	<0.001	(1.56, 3.01)	NA^**^	--	--	--				NA	--	--	--			
No	NA	--	--	--				NA	--	--	--				642	64.5	102	15.9		NS	
Yes (no details collected)	NA	--	--	--				NA	--	--	--				354	35.5	61	17.2			
Reported current substance use																					
None	NA	--	--	--				NA	--	--	--				444	44.6	78	17.6	ref.		
Alcohol	NA	--	--	--				NA	--	--	--				547	54.9	85	15.5		NS	
Marijuana	NA	--	--	--				NA	--	--	--				85	8.5	14	16.5	1.99	0.002	(1.28, 3.08)
Number and type of reported sexual partners in past 12 months (Masaka, Kilifi) or 7 days (Nairobi)																					
None	229	13.8	41	17.9	ref.			326	18.4	34	10.4		NS		109	10.9	21	19.3		NS	
one steady partner	1118	67.2	102	9.1	0.55	<0.001	(0.40, 0.77)	764	43.2	61	8.0				140	14.1	16	11.4			
casual or >1 partner	316	19.0	44	13.9	0.98	0.910	(0.63, 1.51)	241	13.6	24	10.0				747	75.0	126	16.9			
Not asked	0	--	0	--				438	24.8	27	6.2				0	--	0	--			
Report of more than one current sexual partner^***^																					
No, only one	NA	--	--	--				NA	--	--	--				37	3.7	4	10.8		NS	
Yes, more than one	NA	--	--	--				NA	--	--	--				963	96.7	159	16.5			

PR: Prevalence Ratio, CI: Confidence Interval, ref: reference group, NA: data were not collected at this CRC, NS: data were not significant predictors of prevalent HIV in multivariate modeling

^*^ In Masaka condom use was measured with the question: Have you and your partner ever used a condom (yes/no), and if so, how often do you use a condom (Always, more than half of the time, about half of the time, rarely or less than half of the time)? In Kilifi: In the past 12 months, have you used a condom never, sometimes or always during sex? In Kangemi: Have you used a condom before (yes/no)?

^**^ Due to collinearity in the covariates, model failed to converge with discharge and ulcers considered together

^***^ In Kangemi, all volunteers were sexually active, but not all volunteers were sexually active in the past 7 days

Volunteers were followed quarterly or monthly. Cohort sizes were selected to provide a robust estimate of HIV incidence in light of the challenges of recruiting marginalized populations, and ranged from 250–1,000 volunteers. At every visit, volunteers were given HIV counseling and testing by both rapid test and p24 ELISA to detect HIV infection prior to antibody seroconversion (see references in Table 2). Rapid tests were typically performed by venipuncture and done in parallel. Condoms were provided, as was lubricant for the cohorts where anal sex was prevalent. Sexual behavior questionnaires were administered quarterly in Kenya, South Africa, Rwanda and Zambia; and every six months in Masaka. Cohorts with HIV incidence persistently <2% are not presented here (e.g., [19, 20]). Volunteers with incident HIV infection were invited to enroll in the early HIV infection study, 2006–2011, and had their CD4 T cell counts and viral load done at regular intervals. These data were used to inform referrals for antiretroviral therapy provided per the national guidelines. CRCs maintained strong relationships with local health care facilities to assure volunteers would be kept aware of and referred to as needed additional care and treatment as it became available (e.g., medical male circumcision) [21].

**Table 2:** Enrollment and HIV incidence among at-risk volunteers enrolled for HIV prevention preparatory studies stratified by CRC and, where appropriate, sex.

**CRC and study population**	**Enrollment years**	**Year of last study visit**	**Total enrolled**	**Total PY**	**HIV cases detected**	**HIV incidence**	**95% CI**	p value^**^	ref.^***^
HIV discordant couples									
Kigali, Rwanda									Stephenson, 2008; Wall, 2012
DC male negative	2002–11^*^	2011	972	1844	64	3.5	(2.7, 4.4)	0.16	
DC female negative	2002–11^*^	2011	884	1705	45	2.6	(1.9, 3.5)		
Lusaka, Zambia									Kempf, 2008; Stephenson, 2008
DC male negative	1995–11^*^	2011	1656	3367	226	6.7	(5.9, 7.7)	0.003	
DC female negative	1995–11^*^	2011	1393	2848	252	8.9	(7.8, 10.0)		
Ndola, Zambia									Lambdin, 2011
DC male negative	2004–11	2011	416	575	35	6.1	(4.2, 8.5)	0.01	
DC female negative	2004–11	2011	335	399	43	10.8	(7.8, 14.5)		
Masaka, Uganda									Ruzagira, 2011 (a)
DC male negative	2006–11	2011	581	1143	44	3.8	(2.9, 5.2)	0.44	
DC female negative	2006–11	2011	313	514	24	4.7	(3.1, 7.0)		
Masaka, Uganda									Ruzagira, 2011 (a)
DC on ART^∞^	2006–09	2009	152	271	3	1.1	(0, 2.4)	—	
Entebbe, Uganda									NA
DC male negative	2006–09	2010	276	345	5	1.4	(0.6, 3.5)	0.27	
DC female negative	2006–09	2010	322	416	11	2.6	(1.5, 4.8)		
Men who have sex with men									
Kilifi, Kenya	2005–2012^▪^	2013^▪^	726	996	73	7.3	(5.8–9.2)	—	Price, 2012; Sanders, 2013
Nairobi, Kenya	2006–09	2010	303	338	19	5.6	(3.6, 8.8)	—	Price, 2012
Rustenburg, South Africa	2009–10	2011	29	21	2	9.5	(2.4, 38.0)	—	NA
At-risk women									
Cape Town, South Africa									Price, 2012
Younger women^▪▪^	2006–7	2008	337	284	8	2.8	(1.4, 5.6)	—	
Rustenburg, South Africa									
Women, clinic and community based	2008–9	2009	223	67	2	3.0	(0.4, 10.8)	—	Feldblum, 2012
Peripheral community residents	2010–11	2012	323	213	19	8.9	(5.7, 14.0)	—	NA
Kilifi, Kenya									Price, 2012
Female sex workers	2005–2010	2012	315	552	13	2.4	(1.4, 4.1)	—	
Fishing Community members									
Masaka, Uganda									NA
Male	2012–13	2013^▪^	327	244	13	5.3	(3.1, 9.2)	0.10	
Female	2012–13	2013^▪^	217	139	14	10.1	(6.0, 17.0)		
Masaka and Entebbe, Uganda									Seely, 2012
Male	2009–10	2011	450	652	34	4.5	(3.1, 6.7)	0.59	
Female	2009–10	2011	550	554	25	5.2	(3.7, 7.3)		

^*^ IAVI support initiated in 2004

^**^ p value for the incident rate ratio calculator comparing HIV incidence by sex, where study populations are stratified by sex

^***^ For additional details on recruitment, demographics, follow up and earlier reports of HIV incidence. With the exception of Feldblum 2012, all the data above and in Table 3 represents new analyses and additional follow up time.

^▪^ Cohort enrollment currently open

^▪▪^ Recruited women from 16 years old; the only cohort to recruit <18 year olds

^∞^ HIV positive partner in the DC cohort is on ART, too few to analyze by volunteer sex (87 negative men, 65 women)

PY: Person years, IQR: Interquartile range, CI: Confidence Interval, CRC: Clinical Research Center, DC: Discordant Couple (reported sex applies to enrolled HIV uninfected volunteers, HIV infected volunteers were all ART-naive except where noted), NA: reference not available, data not previously published

### Early HIV infection study

The Early HIV Infection Study has been described previously [22, 23]. Briefly, volunteers identified with incident HIV infection were invited to enroll as early as possible (typically within 2 months of their estimated date of infection (EDI)) and follow-up is ongoing. Viral subtype was determined by sequencing the *pol* region of HIV from an early sample [22]. Suspected transmitting partners were invited to enroll, and molecular epidemiologic linkages were determined by sequence comparison of pol, gag, gp120, gp41, and/or long terminal repeat regions [24].

### Data collection and management

Data from the HIV prevalence studies were managed by CRC staff. HIV incidence and early infection study data were managed by the Perinatal HIV Research Unit (PHRU) in Johannesburg, South Africa, except the incidence studies in Rwanda, Zambia, and some in Uganda, which were managed by the CRC staff. Data were transcribed onto a DataFax (Clinical DataFax Systems, Inc., Ontario, Canada) form and digitally faxed to a central server; manual and automatic queries were received then resolved by the CRC data management team. An epidemiologist reviewed the data prior to analysis.

### Data analysis

Inferential statistics include chi square or Wilcoxon rank-sum as appropriate. Predictors of prevalent HIV were evaluated separately for different key populations. Covariates were selected to remain in the multivariable model based on a p-value<0.05. Modeling results are shown as p-values, prevalence ratios, and 95% confidence intervals. Comparisons of HIV incidence across CRC or by gender were done by Cox Proportional Hazards modeling; analyses comparing HIV incidence across time was done using the Stata incidence rate ratio calculator. Only those cohorts with recruitment spread over > 3 years and with adequate follow-up (>400 PY) were analyzed by year. Estimates that vary from published results are due to additional follow-up time included in this report. Data analysis was done with Stata (v12, College Park, TX, USA).

### Ethical approval of clinical research

Prior to enrollment, all volunteers underwent an informed consent procedure and written informed consent was documented. Ethical approvals in each country were obtained from the Kenya Medical Research Institute Ethical Review Committee, the Kenyatta National Hospital Ethical Review Committee of the University of Nairobi, the Rwanda National Ethics Committee, the Uganda Virus Research Institute Science and Ethics Committee, the Uganda National Council of Science and Technology, the University of Cape Town Health Science Research and Ethics Committee, the University of Zambia Research Ethics Committee, the Bio-Medical Research Ethics Committee at the University of KwaZulu Natal, and the Emory University Institutional Review Board.

## Results

### 1. HIV prevalence and associated risk factors in Masaka, Nairobi and Kilifi

From January to December 2004, 4,428 volunteers were enrolled at three CRCs in a cross-sectional study of HIV prevalence. Prevalence varied from 8% to 16%; women and older volunteers had a significantly higher HIV prevalence at each CRC (Table 1). Marital status was associated with HIV infection only at Kilifi, with divorced/widowed and polygamously married volunteers two to three times more likely to be HIV infected than volunteers who had never been married. In Masaka, residing in the community situated along the Kampala-Kigali trans-African highway was associated with a 50% higher HIV prevalence compared to two villages off the highway. In Kilifi, residents of urban areas were four times as likely to be HIV infected as were rural volunteers. In Masaka, inconsistent condom use relative to never using a condom was associated with HIV, and in Nairobi report of ever having used a condom was associated with HIV infection. Report of genital ulcers was associated with a 1.5 to 2.5-fold increase in HIV in Masaka and Kilifi. In Nairobi, marijuana use was associated with a two-fold increased likelihood of HIV infection. In Masaka, report of a single, steady partner was inversely associated with HIV infection relative to report of no sexual partners in the past 3 months.

### 2a. HIV incidence in discordant couples

From 2002 to 2011, 1,856 couples discordant for HIV status were enrolled in Kigali (Table 2). HIV incidence was similar among women and men (p = 0.16). HIV incidence was nearly 2.5 times higher in the first three months of follow-up compared to subsequent visits for men (p = 0.003, Table 3), but for women this only achieved borderline significance. HIV incidence did not drop significantly with calendar year in Kigali (Table 3).

**Table 3:** HIV incidence stratified by duration of time in study follow up, and (where data were available) by study year.

**CRC and study population**	**Total PY**	**HIV cases detected**	**HIV incidence**	**95% CI**	p value^*^
HIV discordant couples					
Kigali, Rwanda: male negative					
First 3 months of follow up	238	17	7.2	(4.2, 11.5)	0.003
>3 months of follow up	1615	47	2.9	(2.1, 3.9)	
2002–2006	947	38	4.0	(2.8, 5.5)	0.20
2007–2011	896	26	2.9	(1.9, 4.3)	
Kigali, Rwanda: female negative					
First 3 months of follow up	219	10	4.6	(2.2, 8.4)	0.08
>3 months of follow up	1494	35	2.3	(1.6, 3.3)	
2002–2006	863	19	2.2	(1.3, 3.4)	0.27
2007–2011	842	26	3.1	(2.0, 4.5)	
Lusaka, Zambia: male negative					
First 3 months of follow up	404	39	9.7	(6.9, 13.2)	0.02
>3 months of follow up	2979	187	6.3	(5.4, 7.2)	
2003–2006	1152	75	6.5	(5.1, 8.2)	0.67
2007–2011	1058	64	6.1	(4.7, 7.7)	
Lusaka, Zambia: female negative					
First 3 months of follow up	341	51	15.0	(11.1, 19.7)	<0.001
>3 months of follow up	2520	201	8.0	(6.9, 9.2)	
–2006	848	91	10.7	(8.6, 13.2)	0.001
2007–2011	741	44	5.9	(4.3, 8.0)	
Ndola, Zambia: male negative					
First 3 months of follow up	99	6	6.1	(2.2, 13.2)	0.96
>3 months of follow up	479	29	6.1	(4.1, 8.7)	
2004–2007	290	23	7.9	(5.0, 11.9)	0.07
2008–2011	284	12	4.2	(2.2, 7.4)	
Ndola, Zambia: female negative					
First 3 months of follow up	79	15	19.1	(10.7, 31.5)	0.02
>3 months of follow up	322	28	8.7	(5.8, 12.6)	
2004–2007	168	23	13.7	(8.7, 20.5)	0.14
2008–2011	231	20	8.7	(5.3, 13.4)	
Masaka, Uganda: male negative (excluding females on ART)					
First 3 months of follow up	127	3	2.4	(0.8, 7.4)	0.39
>3 months of follow up	1017	41	4.0	(3.0, 5.5)	
2006–2008	442	20	4.5	(2.9, 7.0)	0.36
2009–2011	701	24	3.4	(2.3, 5.1)	
Masaka, Uganda: female negative (excluding males on ART)					
First 3 months of follow up	70	4	5.8	(2.2, 15.3)	0.64
>3 months of follow up	445	20	4.5	(2.9, 7.0)	
2006–2008	150	10	6.7	(3.6, 12.4)	0.19
2009–2011	365	14	3.8	(2.3, 6.5)	
Entebbe, Uganda: male negative					
First 3 months of follow up	61	2	3.3	(0.8, 13.2)	0.36
>3 months of follow up	284	4	1.4	(0.5, 3.7)	
Entebbe, Uganda: female negative					
First 3 months of follow up	72	2	2.8	(0.7, 11.1)	0.89
>3 months of follow up	344	9	2.6	(1.4, 5.0)	
Men who have sex with men					
Nairobi, Kenya					
First 3 months of follow up	64	5	7.8	(3.2, 18.7)	0.42
>3 months of follow up	274	14	5.1	(3.0, 8.6)	
Kilifi, Kenya					
First 3 months of follow up	94	15	16.0	(9.6, 26.5)	<0.001
>3 months of follow up	829	43	5.2	(3.8, 7.0)	
2005–2008	304	25	8.2	(5.6–12.2)	0.49
2009–2012	692	48	6.9	(5.2–9.2)	
At-risk women					
Cape Town, South Africa: Sexually active women					
First 3 months of follow up	76	4	5.2	(2.0, 14.0)	0.18
>3 months of follow up	207	4	1.9	(0.7, 5.1)	
Rustenburg, South Africa: Sexually active women					
First 3 months of follow up	65	8	12.3	(6.2, 24.7)	0.28
>3 months of follow up	149	11	7.4	(4.1, 13.4)	
Fishing Community members					
Masaka, Uganda (2012–2013 cohort)					
First 3 months of follow up	119	9	7.6	(3.9,14.6)	0.25
>3 months of follow up	383	18	4.7	(3.0,7.5)	
Masaka & Entebbe, Uganda (2009–2010 cohort)					
First 6 months of follow up^**^	417	23	5.5	(3.7, 8.3)	0.28
>6 months of follow up	803	33	4.1	(2.9, 5.8)	

^*^ Comparing HIV incidence in the first three months to HIV incidence in subsequent months of follow up and HIV incidence in the first set of calendar years indicated to the subsequent set of years, where data were available

^**^ First return visit for this cohort was at six months post enrollment

From 1995 (2004 in Ndola) to 2011, 3,800 HIV-discordant couples were enrolled at two CRCs in Zambia (Table 2). HIV incidence was similar across the two CRCs (p = 0.71) and was significantly higher in women compared to men in both Lusaka (p = 0.003) and Ndola (p = 0.01). HIV incidence in the first three months of study follow-up was significantly higher than in subsequent visits for women at both CRCs (Table 3), but the same was true only for men in Lusaka. With the exception of Lusaka women, HIV incidence did not vary significantly by year (Table 3), although a trend was also borderline significant among men in Ndola.

From 2006 through 2011, 1,574 couples were enrolled at both Ugandan CRCs, including 152 couples in Masaka who contributed follow-up while the HIV infected partner was on ART (Table 2). HIV incidence did not vary by sex (Table 2) but was significantly lower in Entebbe than in Masaka (p = 0.02). Masaka was the only CRC that enrolled discordant couples with the positive partner on ART, and HIV incidence was significantly lower in couples on ART compared to those not on ART (p = 0.009). In 2009, enrollment criteria changed in Masaka. There was no evidence of a drop in HIV incidence by time on study (Entebbe and Masaka) or by calendar year (Masaka) (Table 3).

### 2b. HIV incidence in men who have sex with men

In August 2005, the Kilifi, Kenya, CRC enrolled the first MSM into their HIV incidence cohort study. Through December 2012, 726 MSM have been enrolled in Kilifi and enrollment is ongoing; from January 2006-October 2009, 303 were enrolled in Nairobi; and from April 2009-November 2010, 29 in Rustenburg, South Africa. HIV incidence has been very high in these cohorts, from 5–10 cases/100 person-years (Table 2). Incidence dropped three-fold after the first three months on-study in Kilifi (Table 3, p<0.001); no similar drop was observed in Nairobi. No drop was observed in HIV incidence by calendar time (Kilifi).

### 2c. HIV incidence in Ugandan fishing communities

Between February and August 2009, 1,000 volunteers were enrolled from five fishing communities near Entebbe and Masaka, Uganda, into an 18-month prospective study. The overall HIV incidence was 4.9 cases/100 PY and did not vary by sex (Table 2) or by recruitment area. No evidence for a reduction in HIV incidence with time on study was observed (Table 3). Relatively low HIV incidence was observed among adolescents (ages 13–17: 2.8 cases/100 PY), however few enrolled (n = 26). Using updated screening criteria based on these results, a further 529 volunteers were enrolled in the Masaka district from January 2012; follow-up is ongoing and HIV incidence is high (6.7 cases/100 PY), particularly among women (Table 2).

### 2d. HIV incidence in other key populations

Other cohorts include female sex workers (FSW) in Kilifi [20], younger women in Cape Town [20], and women enrolled from the community and local clinics in Rustenburg [25] (Table 2). In 2010, the Rustenburg CRC shifted recruitment strategies into poorer, peripheral communities where HIV prevalence was higher. Eligibility criteria were also changed to include any woman reporting sexual activity or an STI. Over 300 women enrolled in Rustenburg with these eligibility criteria had an HIV incidence of nearly 9 cases/100PY (Table 2).

### 2. Epidemiologic linkages in the early HIV infection cohort

From February 2006 through December 2011, 613 volunteers with incident HIV infection were enrolled. The cohort comprised 255 (41.5%) women and 359 (58.5%) men, of whom 92 (25.6%) were MSM; all but three from Kenya. Most volunteers (460, 75.0%) were enrolled from HIV-discordant couples cohorts, 219 (47.6%) were women.

For 407 volunteers, suspected transmitting partners were enrolled, 393 (96.6%) of whom had sample to test HIV transmission linkages. The proportion of volunteers linked by HIV sequence similarity to their suspected transmitting partner ranged from 67.1% in the Copperbelt (Kitwe and Ndola) to 100% in Entebbe but these differences did not quite achieve significance (Table 4, p = 0.08, Fisher’s exact test), and they did not vary by the sex of the incident case (p = 0.27). In Kigali, a non-significant trend suggested women with incident HIV were more commonly linked to their partners than were men (36/41 [87.8%] vs. 38/52 [73.1%], respectively, p = 0.08).

**Table 4:** Number of volunteers with HIV sequence data who enrolled with their suspected partner and were epidemiologically linked.

	**Enrolled**	**Linked**		
	**N**	**N**	**%**	**p value**
Total	393	282	71.8	
RC and country				
Kigali, Rwanda	93	74	79.6	0.08^*^
Lusaka, Zambia	145	100	70.0	
Copperbelt, Zambia	76	51	67.1	
Kilifi, Kenya	4	3	75.0	
Entebbe, Uganda	12	12	100.0	
Masaka, Uganda	63	42	66.7	
Sex of incident case				
Male	220	153	69.5	0.27
Female	173	129	74.6	

RC: Research Center

^*^Fisher’s exact test

## Discussion

From 2004 to present, IAVI has coordinated HIV observational epidemiology studies at nine CRCs in preparation for HIV prevention trials. Laboratory, clinical, and data management procedures were standardized to ensure comparability across CRCs. Research protocols typically began with 1) cross-sectional HIV prevalence research, followed by 2) HIV incidence cohorts, and then 3) early HIV infection cohorts. 4,428 volunteers were initially enrolled in Kenya and Uganda, and HIV prevalence was observed to be higher than the national average, even in the general population survey in Masaka. Nine CRCs enrolled over 10,000 HIV-negative volunteers in prospective studies of HIV incidence, which ranged from 2% to 10% annually. This work included the first prospective studies of HIV incidence in African MSM [20, 26]. Over 600 volunteers with incident infection were enrolled from these cohorts into the early HIV infection study. Among those with an identified HIV-positive regular sexual partner, about 28% of HIV transmissions were not from that partner.

Prospective studies remain the gold standard for obtaining reliable HIV incidence data [6] needed for informed decision making, from national level program planning to clinical trial design. However, prospective incidence studies are expensive, time consuming, and require real-time review of data to maintain focus on a study cohort that reflects true risk of HIV infection. While a goal of the 2004 prevalence studies was to guide selection of key populations, some populations with elevated HIV prevalence had low HIV incidence. This included data from rural villages in Masaka district [19] where a previous HIV prevention trial had been conducted [27], heterosexual men in Kenya [20], FSW in Nairobi [20] and HIV discordant couples in Kilifi (no observed HIV incidence, unpublished data). In Masaka, the investigative team shifted focus to HIV discordant couples and, later, to residents of fishing communities. In Kenya the focus changed to MSM when men came forward asking to enroll as sex workers. Among MSM along the coast, the Kilifi team recorded HIV incidence as high as 35 cases/100PY among those who report sex exclusively with other men [26] highlighting both the success of careful epidemiology and the extreme need for intervention. Collateral benefits from our work in MSM and sex workers include the development of health care provider training modules to strengthen service delivery to these marginalized groups [15], and the improvement of our outreach and educational activities within the larger community. Following an attack on our Kilifi clinic that took place in 2010 [28] an emphasis was placed on engaging a wide range of public officials and public health stake holders, from religious leaders to government and police officials, and a community liaison officer has been hired whose role is to manage this process of outreach and education. Each CRC successfully identified key populations both suitable for HIV prevention trials and in need of specialized program planning to reduce HIV transmission [15–17].

Among those cohorts where higher risk was observed, we recorded heterogeneity associated with volunteer sex, time on study, and calendar year. Women are frequently cited as at significant risk for HIV acquisition in Africa [29], and in five of the seven cohorts with men and women the incidence point estimate reflected this (Table 2), although this achieved statistical significance only in Zambia. Of the more than a dozen cohorts presented in Table 3, five (men in discordant couples at Lusaka, Ndola, and Kigali; women in Ndola; and MSM in Kilifi) recorded a reduction in HIV incidence after the first study follow-up visit. While pre-seroconversion HIV infection at enrollment may artificially inflate HIV incidence at the first follow-up visit, we employed the p24 antigen assay at every study visit thereby excluding some volunteers at enrollment prior to antibody seroconversion. Nine cohorts in table 3 had adequate follow-up time to consider HIV incidence as a function of calendar year. Only female members of DCs in Lusaka showed a statistically significant reduction in HIV incidence. Of the 16 cohorts reported in Table 3, 5 demonstrate a significant drop in HIV incidence, 9 show a non-significant drop in the incidence point estimate, 1 shows no change, and 1 shows a non-significant increase in the point estimate. We feel the most likely reason for the drop in HIV incidence was the regular counseling and testing and prevention messages, however, some cohorts were simply not powered to detect this drop (e.g., MSM in Nairobi, women in Cape Town and Rustenburg saw a non-significant drop of ~3 cases per 100 person years. This would otherwise represent a very large drop, but in our case, it remains non-significant in a 200–300 person cohort). Additionally, the different stages of the epidemic in different countries and cohorts may explain why we observe no significant changes in incidence, as perhaps some mature epidemics may be less susceptible to intervention. Cohort recruitment may change subtly over time as new clients seek HIV testing at the CRCs. Secular trends in the source populations such as increased awareness of HIV prevention techniques may also be prevalent. Counseling and prevention messages improve as health care providers get to know their stakeholders better. Treatment and care changes as antiretroviral therapy guidelines change, affecting risk of HIV acquisition in certain populations (e.g., HPTN 052 and the recommendation that ART be initiated in HIV-discordant couples). National level program planning requires incidence trends over time to best assess whether interventions are working or not. Clinical trial design (particularly sample size) should always factor in anticipated decline in incidence during the time of study as a result of national program interventions and that of the study itself. Taken as a whole, these data suggest that in our cohorts HIV incidence tends to be lower in men than women, declines with time on study, and may drop with calendar time. It is with caution that one should generalize findings in research cohorts to the broader community; however this is similar to overall trends in the African epidemic [2, 29].

Volunteers with incident HIV infection from our discordant couple cohorts provide an interesting lesson on risk that applies both to the clinical trialist and the public policy maker. Some of the highest HIV incidence was observed in these cohorts, with incidence estimates up to 10 cases /100PY. The HIV incidence among discordant couples who are not receiving quarterly behavioral counseling interventions is likely to be higher—possibly as high as 20–25% annually [30, 31]. Following the results of HPTN 052, which demonstrated a 96% reduction of HIV transmission within discordant couples when the volunteer with HIV infection received ART early [32], considerable attention has been focused on “test and treat” (i.e., putting newly diagnosed HIV-positive persons on ART as quickly as possible) as a means to reduce HIV transmissions [33, 34], and discordant couples represent a priority [35]. While others point out that real-world adherence, funding, and logistics all provide significant hurdles to this lofty goal [36], our data highlight another challenge: up to 30% of infections in our discordant couple cohorts originated from a partner other than the HIV infected steady partner. Indeed, in HPTN 052, 7 (17.9%) of the 39 transmission events were linked to someone other than the volunteer’s enrolled HIV-positive partner [32]. Although “test and treat” may eventually be a practical reality for much of the world’s population living with HIV, until that time discordant couples remain an important key population for public health interventions and for study.

We enrolled thousands of volunteers at risk for HIV infection, hundreds of volunteers with incident HIV infection, and identified key populations in need of public health interventions who might be suitable to participate in the next generation of HIV prevention trials. This work has spearheaded improvements to the health care delivery for marginalized groups such as sex workers and MSM [15] and reinforced the importance of testing and counseling couples together [30, 37, 38]. Specimens from these studies have also been the basis for dozens of ongoing collaborations ranging from estimating the seroprevalence of potential vaccine vectors [39] to characterizing the transmitted virus [40]and the host immune system following infection [41, 42]. IAVI has also tested 13 HIV vaccine products in 25 early-phase clinical trials in Africa [43–47], conducted a multi-site study to establish adult safety laboratory reference ranges against which to characterize adverse events in these clinical trials [48], and enrolled over 1,800 asymptomatic volunteers with prevalent HIV infection in a search for broadly neutralizing antibodies [49]. Collateral benefits extend beyond these CRCs as GCLP and assay-specific training are made available to laboratory technicians outside this partnership.

These large prospective observational epidemiology studies provide valuable data for prevention trial design and conduct, prevention planning, and service delivery. Over the past nine years, IAVI has identified and enrolled thousands of volunteers from key populations, observing a high HIV incidence despite regular HIV testing and counseling. The benefits to the public health, scientific and local communities are tremendous.
